# Supplementary material for: Effects of general and central adiposity on circulating lipoprotein, lipid, and metabolite levels in UK Biobank: A multivariable Mendelian randomization study
Source: Lancet Reg Health Eur. 2022 Jul 6;21:100457. doi: 10.1016/j.lanepe.2022.100457 (PMC9272390; doi:10.1016/j.lanepe.2022.100457)
Supplement: Supplementary file 1 [file mmc1.pdf]

Supplementary Figure 1 Conventional observational estimates of the total (unadjusted) and direct (adjusted) effects of BMI and WHR on lipoproteins, cholesterol, and triglycerides measured using NMR among 89,540 UK Biobank participants

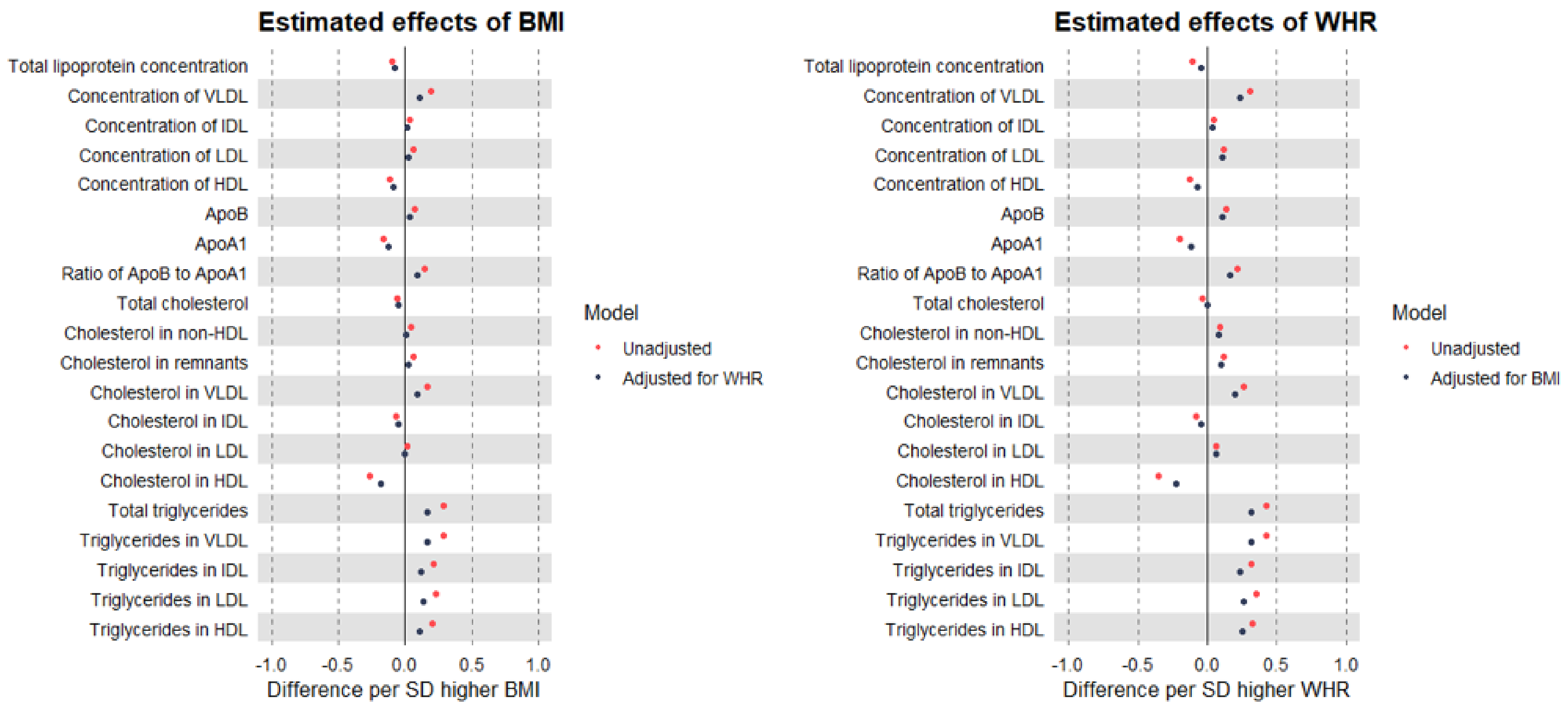

Estimates are standardised betas and 95% confidence intervals (not visible due to small standard errors). ApoB: Apolipoprotein B. ApoA-1: Apolipoprotein A-1. VLDL: Very-low-density lipoprotein. IDL: Intermediate-density lipoprotein. LDL: Low-density lipoprotein. HDL: High-density lipoprotein.

Supplementary Figure 2 Conventional observational estimates of the total (unadjusted) and direct (adjusted) effects of BMI and WHR on selected metabolites measured using NMR among 89,540 UK Biobank participants

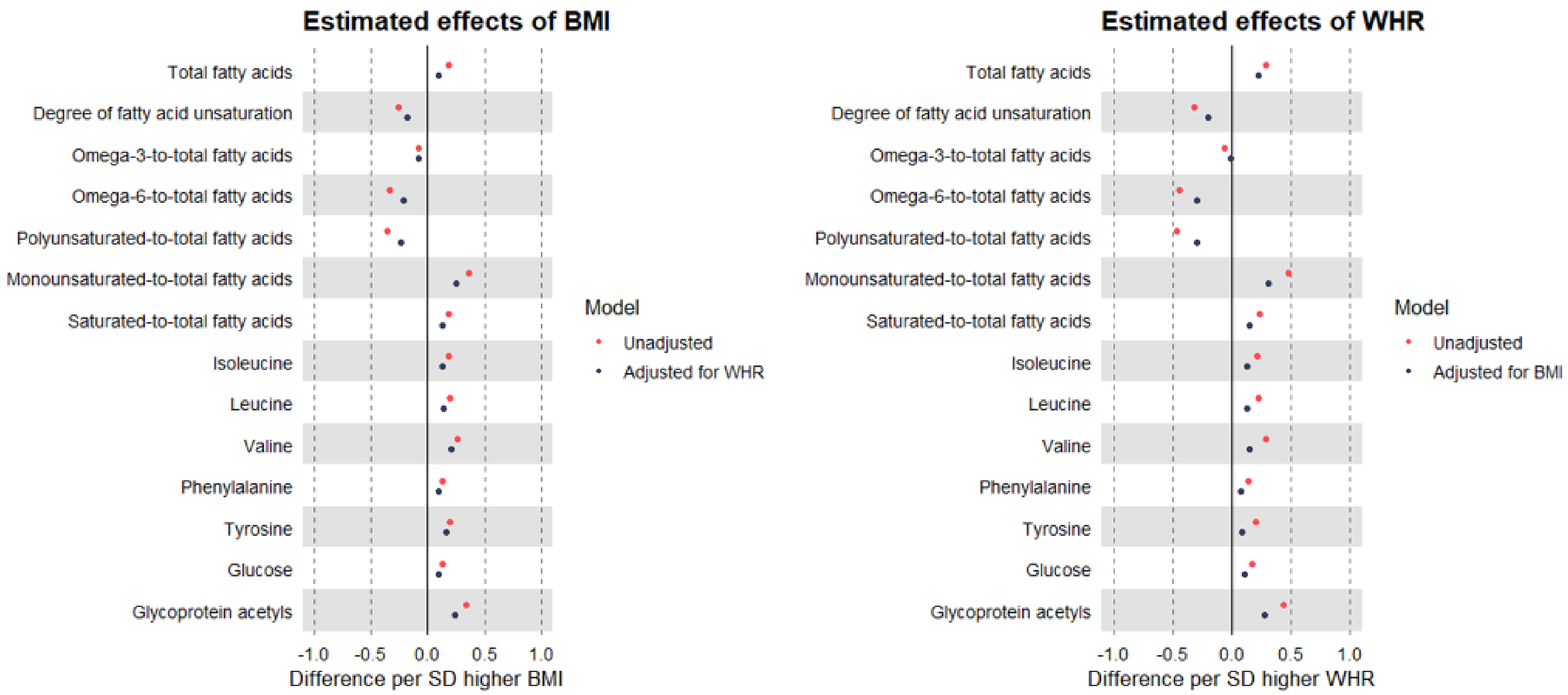

Estimates are standardised betas and 95% confidence intervals (not visible due to small standard errors).

Supplementary Figure 3 Sex-specific conventional observational estimates of the total (unadjusted) and direct (adjusted) effects of BMI and WHR on lipoproteins, cholesterol, and triglycerides measured using NMR among 89,540 UK Biobank participants

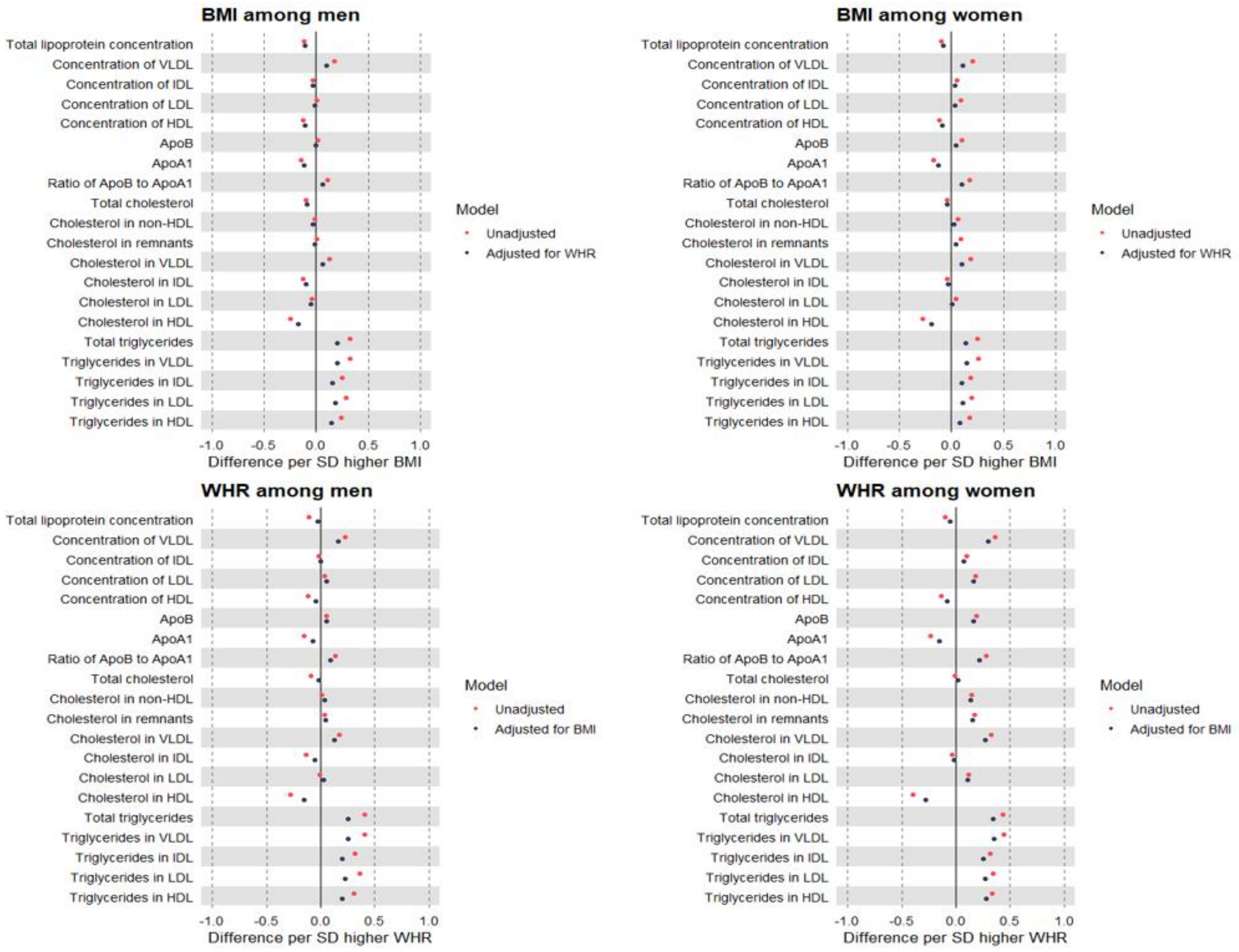

Estimates are standardised betas and 95% confidence intervals (not visible due to small standard errors). ApoB: Apolipoprotein B. ApoA-1: Apolipoprotein A-1. VLDL: Very-low-density lipoprotein. IDL: Intermediate-density lipoprotein. LDL: Low-density lipoprotein. HDL: High-density lipoprotein.

Supplementary Figure 4 Sex-specific conventional observational estimates of the total (unadjusted) and direct (adjusted) effects of BMI and WHR on selected metabolites measured using NMR, among 89,540 UK Biobank participants

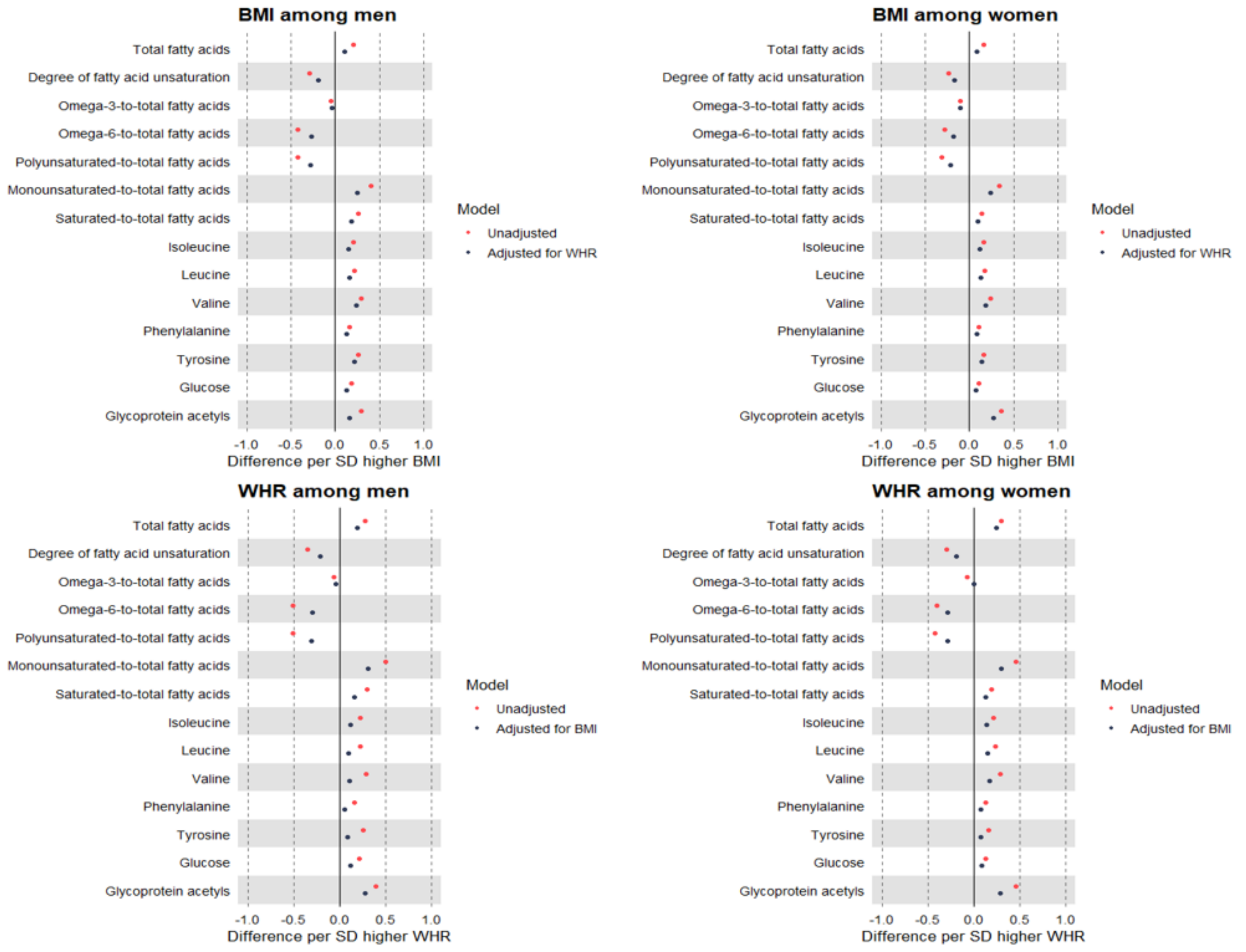

Estimates are standardised betas and 95% confidence intervals (not visible due to small standard errors). ApoB: Apolipoprotein B. ApoA1: Apolipoprotein A1. VLDL: Very-low-density lipoprotein. IDL: Intermediate-density lipoprotein. LDL: Low-density lipoprotein. HDL: High-density lipoprotein.

Supplementary Figure 5 Conventional observational estimates of the direct (mutually adjusted) effects of BMI and WHR on non-HDL cholesterol measured using NMR, by age tertile as a proxy for medication use among 89,540 UK Biobank participants

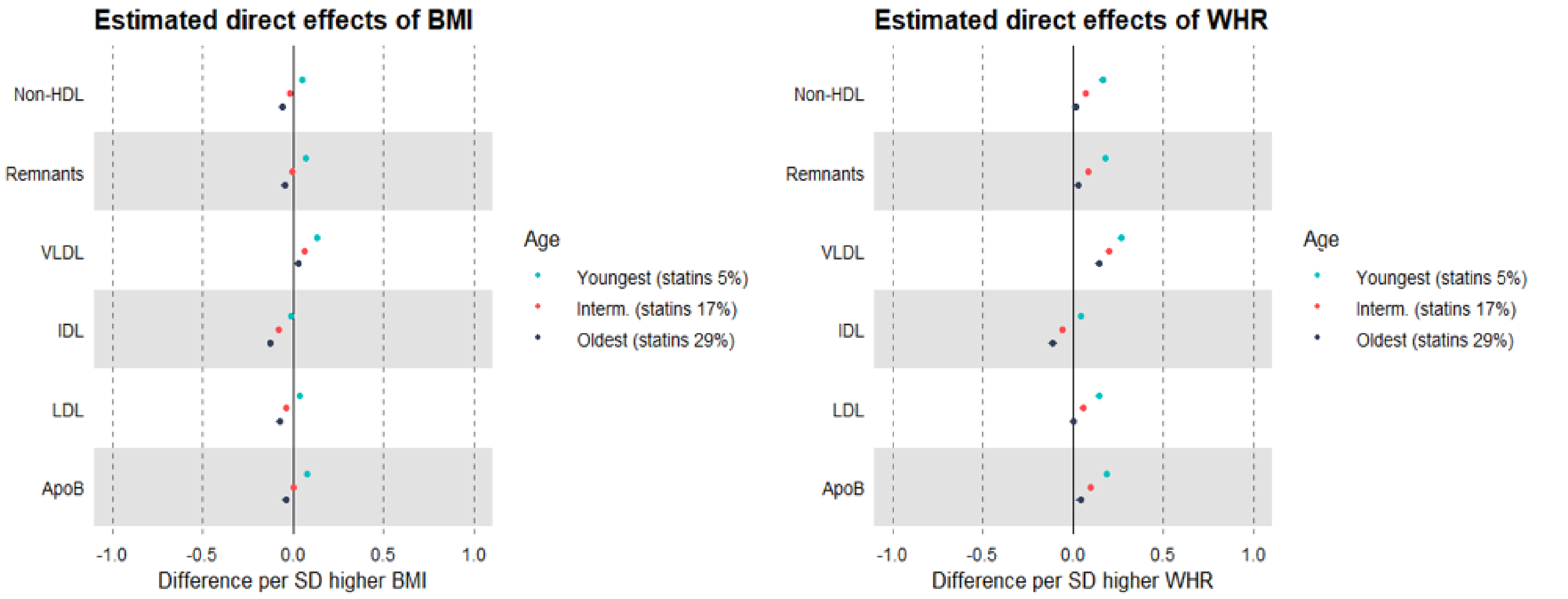

Estimates are standardised betas and 95% confidence intervals (not visible due to small standard errors). HDL: High-density lipoprotein. VLDL: Very-low-density lipoprotein. IDL: Intermediate-density lipoprotein. LDL: Low-density lipoprotein. ApoB: Apolipoprotein B.

Supplementary Figure 6 Sex-specific conventional observational estimates of the direct (mutually adjusted) effects of BMI and WHR on non-HDL cholesterol measured using NMR, by age tertile as a proxy for medication use among 89,540 UK Biobank participants

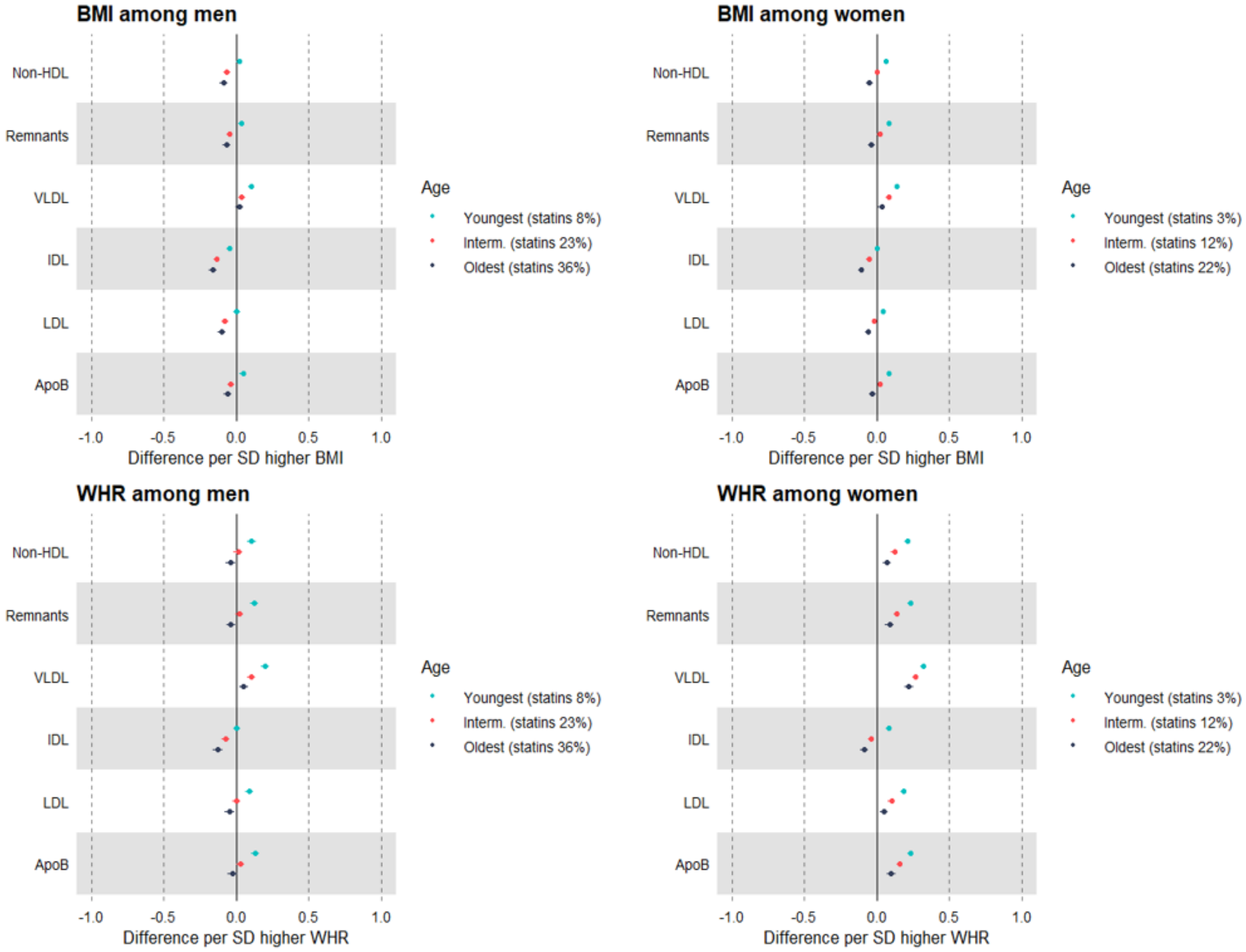

Estimates are standardised betas and 95% confidence intervals (not visible due to small standard errors). HDL: High-density lipoprotein. VLDL: Very-low-density lipoprotein. IDL: Intermediate-density lipoprotein. LDL: Low-density lipoprotein. ApoB: Apolipoprotein B.
